# Efficacy of Early Combination Therapy With Lianhuaqingwen and Arbidol in Moderate and Severe COVID-19 Patients: A Retrospective Cohort Study

**DOI:** 10.3389/fphar.2020.560209

**Published:** 2020-09-18

**Authors:** Jie Fang, Hui Li, Wei Du, Ping Yu, Ying-Yun Guan, Shi-Yu Ma, Dong Liu, Wei Chen, Guo-Chao Shi, Xiao-Lan Bian

**Affiliations:** ^1^ Department of Pharmacy, Ruijin Hospital, School of Medicine, Shanghai Jiaotong University, Shanghai, China; ^2^ Department of Respiration and Critical Care Disease, Ruijin Hospital, School of Medicine, Shanghai Jiaotong University, Shanghai, China; ^3^ School of Medicine, Institute of Respiratory Diseases, Shanghai Jiaotong University, Shanghai, China

**Keywords:** COVID-19, SARS-CoV-2, Arbidol, Lianhuaqingwen, combination therapy

## Abstract

**Objective:**

Since the outbreak of severe acute respiratory syndrome coronavirus 2 (SARS-CoV-2) in Wuhan City, China, coronavirus disease 2019 (COVID-19) has become a global pandemic. However, no special therapeutic drugs have been identified for COVID-19. The aim of this study was to search for drugs to effectively treat COVID-19.

**Materials and Methods:**

We conducted a retrospective cohort study with a total of 162 adult inpatients (≥18 years old) from Ruijin Hospital (Shanghai, China) and Tongji Hospital (Wuhan, China) between January 27, 2020, and March 10, 2020. The enrolled COVID-19 patients were first divided into the Lianhuaqingwen (LHQW) monotherapy group and the LHQW + Arbidol combination therapy group. Then, these two groups were further classified into moderate and severe groups according to the clinical classification of COVID-19.

**Results:**

The early combined usage of LHQW and Arbidol can significantly accelerate the recovery of patients with moderate COVID-19 by reducing the time to conversion to nucleic acid negativity, the time to chest CT improvement, and the length of hospital stay. However, no benefit was observed in severe COVID-19 patients treated with the combination of LHQW + Arbidol. In this study, both Arbidol and LHQW were well tolerated without serious drug-associated adverse events.

**Conclusion:**

The early combined usage of LHQW and Arbidol may accelerate recovery and improve the prognosis of patients with moderate COVID-19.

## Introduction

In December 2019, the severe acute respiratory coronavirus 2 (SARS-CoV-2) outbreak began in Wuhan, China. The respiratory disease caused by this virus was termed coronavirus disease 2019 (COVID-19), and it has spread rapidly, becoming a worldwide pandemic (Pedersen and Ho, 2020). As of June 7, 2020, COVID-19 had spread to 215 countries, with 6.79 million confirmed cases and 39.7 thousand confirmed deaths (World Health Organization, 2020). COVID-19 is an acute respiratory tract infection that can lead to the development of acute respiratory distress syndrome. The most common symptoms are fever and cough, while diarrhea is rare. Lymphocytopenia and ground-glass opacity on the chest computed tomography (CT) are typical characteristics of COVID-19 in China (Guan et al., 2020). Older age (≥65 years); preexisting concurrent cardiovascular or cerebrovascular disease; higher temperature; higher blood leukocyte count, CD3^+^CD8^+^ T cell count, neutrophil count, and neutrophil percentage; higher levels of C-reactive protein (CRP), D-dimer, lactate dehydrogenase (LDH), creatine kinase and cardiac troponin; and higher activities of alanine aminotransferase, aspartate aminotransferase, and *α*-hydroxybutyrate dehydrogenase are closely related to severe COVID-19 and the composite endpoint (Zhang et al., 2020; Zhou et al., 2020; Du et al., 2020). In one single-center case study of 138 hospitalized patients with confirmed COVID-19 in Wuhan, the overall mortality rate was 4.3% (Wang D. et al., 2020). To date, this infectious disease has been controlled in China. However, it continues to spread, and the number of confirmed cases is still increasing in other countries. Consequently, specific vaccines and effective antiviral drugs are urgently needed.

Undoubtedly, great efforts have been made to identify effective medical products, such as drugs and vaccines, to diagnose and treat COVID-19 patients. To our knowledge, there are still no specific therapeutic drugs for COVID-19. However, some drugs have been reported to be effective in clearing SARS-CoV-2 or improving symptoms. For example, the most promising antiviral drug, remdesivir, resulted in clinical improvement in 36 of 53 patients (68%) with severe COVID-19, indicating its potential effectiveness (Grein et al., 2020). However, the efficacy and safety of remdesivir still need to be assessed in randomized controlled trials. However, there were some drugs that were expected to be useful that were shown to be ineffective. The addition of the antiviral drug lopinavir–ritonavir to standard supportive care had no benefit in terms of clinical improvement or mortality in seriously ill patients with COVID-19 compared with the effects of standard care alone (Cao et al., 2020).

In the early stage of the COVID-19 outbreak, Chinese scientists found that Arbidol, a broad-spectrum antiviral drug that was first developed by the Russian Research Chemical-Pharmaceutical Institute, could effectively inhibit SARS-CoV-2 infection at a concentration of 10–30 μM *in vitro* (Global Times, 2020). This suggests that Arbidol may have the ability to inhibit SARS-CoV-2 *in vivo*, making it a promising therapeutic drug for COVID-19. One clinical study found that COVID-19 patients in the Arbidol group had a shorter duration of positivity on RNA tests than those in the lopinavir/ritonavir group (Zhu Z. et al., 2020). Four clinical trials were conducted to explore the efficacy of Arbidol for the treatment of COVID-19 (Rosa and Santos, 2020). Based on historical records of SARS and H1N1 influenza treatment, traditional Chinese medicine (TCM) could also be an alternative approach to the prevention and treatment of COVID-19 (Luo et al., 2020; Yang et al., 2020). A retrospective cohort study in COVID-19 patients also revealed that LHQW combined with routine treatment significantly relieved the primary symptoms and shortened the disease course (Yao and Li, 2020). Arbidol and a variety of TCMs (LHQW, Shufengjiedu and Chinese herbal formulae) were all recommended by the Chinese guidelines for the diagnosis and treatment of COVID-19, which are issued by the National Health Commission of the People’s Republic of China. One case report of four patients with mild or severe COVID-19 in Shanghai (China) found that combining antiviral drugs (lopinavir/ritonavir or Arbidol) with TCM (Shufengjiedu capsule) resulted in a significant improvement in clinical symptoms (Wang Z. et al., 2020). However, the efficacy of combining antiviral drugs with TCM still needs further verification in clinical trials. Therefore, we conducted this retrospective study to evaluate the efficacy of LHQW monotherapy and LHQW + Arbidol combination therapy for COVID-19.

## Methods

### Study Design and Participants

Adult inpatients (≥18 years old) from Ruijin Hospital (Shanghai, China) and Tongji Hospital (Wuhan, China) were recruited for this retrospective study. The recruitment period was from January 27, 2020, to March 10, 2020. All patients enrolled in this study were diagnosed with COVID-19 with a real-time reverse-transcriptase polymerase chain reaction (RT-PCR) assay of nasal and pharyngeal swab specimens and CT image features of pneumonia according to the diagnostic criteria of the guidelines for the diagnosis and treatment of COVID-19 in China. Outcomes were followed up until March 23, 2020. Patients meeting any of the following criteria were excluded: 1) patients with critical underlying diseases who were transferred to another hospital; 2) patients with severe underlying disease who were transferred to the ICU or died within 48 h after admission; 3) patients who were not treated with Arbidol or LHQW; and 4) patients who lacked important data. A total of 162 of the 178 patients met the inclusion criteria and were enrolled in this study.

### Procedures

**Figure 1:**
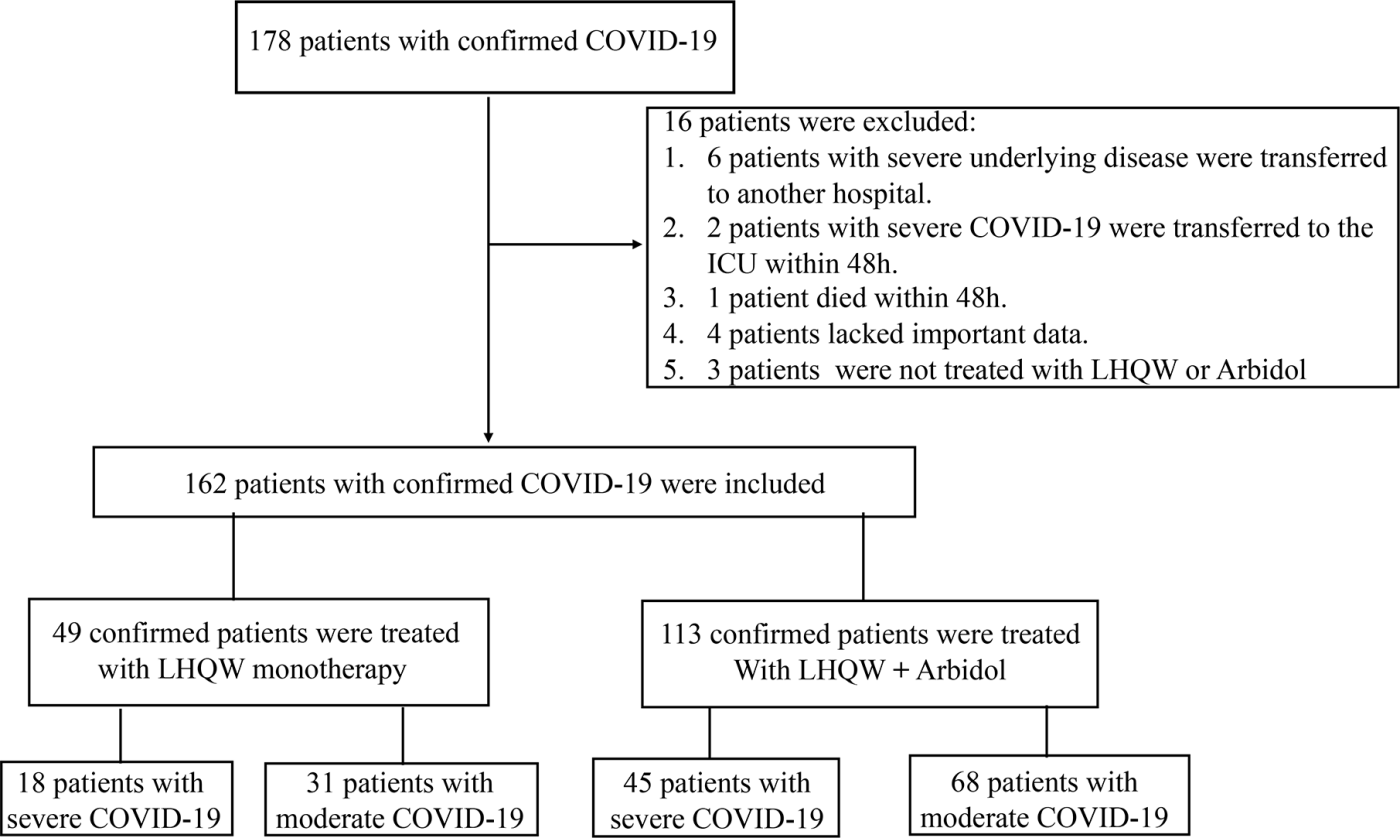
The flow diagram of this study.

Based on the therapeutic drugs administered, the patients were first divided into the LHQW monotherapy group and the LHQW **+** Arbidol combination therapy group. Both the LHQW group and the LHQW **+** Arbidol group included moderate and severe COVID-19 patients. Then, these two groups were further classified into moderate and severe groups according to the clinical classification of COVID-19. The detailed flow diagram of this study is shown in **Figure 1**. The LHQW monotherapy group was treated with LHQW capsules (four capsules each time, three times a day). The LHQW **+** Arbidol combination therapy group was simultaneously treated with LHQW capsules (four capsules each time, three times a day) **+** Arbidol tablets (200 mg each time, three times a day). After treatment for 7 days, SARS-CoV-2 nucleic acid tests and CT imaging examinations were carried out to evaluate the therapeutic effect.

### Data Collection

The medical records of COVID-19 patients were reviewed, and the demographic, clinical laboratory, treatment, and outcome data were extracted from the electronic medical records. Two physicians (WD and WC) checked all the data, and two clinical pharmacists (JF and HL) adjudicated any differences in interpretation.

### Definitions

#### COVID-19 Clinical Classification

According to the fifth edition of the guidelines for the diagnosis and treatment of COVID-19, moderate and severe COVID-19 were defined as follows:

1. Moderate COVID-19
  Fever and respiratory symptoms, such as cough, were present and pneumonia was indicated on chest CT.
2. Severe COVID-19
  Adult patients with one of the following conditions were classified as having severe COVID-19:
  - Respiratory distress, respiratory rate ≥30 per minute;
  - Oxygen saturation ≤93% on ambient air at rest;
  - Partial pressure of oxygen in arterial blood/fraction of inspired oxygen ≤300 mmHg.

The conversion of nucleic acid to negative was defined as two consecutive negative results of RT-PCR for SARS-CoV-2 RNA in nasal and pharyngeal swab specimens; the two nucleic acid tests were conducted least 24 h apart. Improvement on chest CT was defined when the extent of lung involvement decreased by more than 10%, and the severity of pneumonia was alleviated. The extent of lung involvement was defined as the area of consolidation and/or patchy opacity and ground-glass opacity in the lungs. The times to nucleic acid conversion to negativity/chest CT improvement were defined as the time from the confirmation of the diagnosis of COVID-19 to the first negative nucleic acid test/chest CT with improvement. The length of hospital stay was defined as the time from admission to discharge. The time from illness onset to antiviral therapy was defined as the time from illness onset to treatment with LHQW or LHQW **+** Arbidol. Fever was defined as an axillary temperature of at least 37.3℃. Consolidation was defined as opacification with obscuration of the margins of vessels and airway walls. Ground-glass opacity was defined as hazy increased lung attenuation with the preservation of bronchial and vascular margins (Hansell DM and MacMahon, 2008). Medication history was defined as drugs taken by patients from the time of symptom onset with the purpose of treating COVID-19. Early LHQW monotherapy and LHQW **+** Arbidol combination therapy were defined as an interval from illness onset to the beginning of therapy ≤4 days. Non-early LHQW monotherapy and LHQW **+** arbidol combination therapy were defined as an interval from illness onset to the beginning of combination therapy >4 days.

### Statistical Analysis

Statistical analyses were performed with SPSS software (version 25; SPSS Inc., Chicago, IL, USA). Continuous variables are presented as the medians (interquartile range, IQR) or means ± SEMs. Student’s t test was used to analyze samples with a normal distribution, and the Mann–Whitney U test was used to analyze samples with a non-normal distribution. Categorical variables are expressed as n (%) and were analyzed using the chi-square test or Fisher’s exact test to compare differences, as appropriate. The survival analysis was performed with a log-rank test. Linear regression was used to analyze the correlation between the time to conversion to nucleic acid negativity, time to chest CT improvement, duration of hospital stay and time from illness onset to LHQW **+** Arbidol combination therapy. For all statistical tests, a P value <0.05 (bilateral) was considered statistically significant.

## Results

### Comparison of LHQW Monotherapy and LHQW + Arbidol Combination Therapy Among All Enrolled COVID-19 Patients

**Figure 2:**
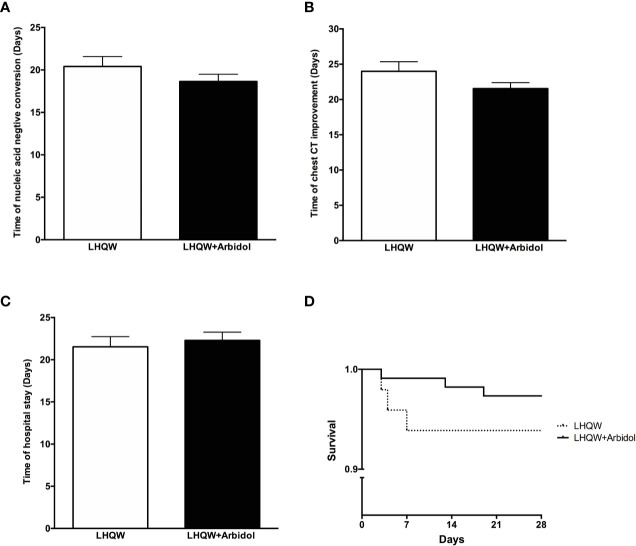
Efficacy of LHQW and LHQW + Arbidol treatment among all enrolled COVID-19 patients. **(A)** Effect on the time to conversion to nucleic acid negativity. **(B)** Effect on time to chest CT improvement. **(C)** Effect on length of hospital stay. **(D)** Comparison of Kaplan–Meier survival curves of LHQW and LHQW + Arbidol treatment in all enrolled COVID-19 patients.

A total of 162 eligible patients with COVID-19 were recruited from two hospitals in Wuhan and Shanghai cities for the full analysis: 49 patients were in the LHQW monotherapy group, and 113 patients were in the LHQW **+** Arbidol combination therapy group. The detailed analysis of the demographic, clinical, laboratory, and radiographic characteristics and the medication history of all enrolled patients is summarized in **Table 1**. No significant differences in the demographic, clinical, laboratory, and radiographic characteristics or treatments were found between the LHQW monotherapy group and the LHQW **+** Arbidol combination therapy group. We also found that there were no significant differences in the time to conversion to nucleic acid negativity, time to chest CT improvement, length of hospital stay or Kaplan–Meier survival curves between the LHQW group and the LHQW **+** Arbidol group (**Figure 2**). Therefore, in the subsequent analyses, we investigated the efficacy of LHQW and LHQW **+** Arbidol treatments in patients with severe and moderate COVID-19.

**Table 1:** Demographic, clinical, laboratory, and radiographic characteristics and the medication history of all enrolled patients with COVID-19.

	Total	LHQW	LHQW + Arbidol	p value
	(n = 162)	(n = 99)	(n = 63)	
**Demographic and clinical characteristics**				
Age	61.5 (50–69)	65 (55.5–74)	63 (54.75–71)	0.491
Sex	–	–	–	0.206
Female	75 (46.3%)	19 (38.8%)	56 (49.6%)	–
Male	87 (53.7%)	30 (61.2%)	57 (50.4%)	–
Hypertension	64 (39.5%)	19 (38.8%)	45 (39.8%)	0.9
Diabetes	22 (13.7%)	7 (14.3%)	15 (13.4%)	0.741
Chronic obstructive lung disease	10 (6.2%)	4 (8.2%)	6 (5.3%)	0.492
Coronary heart disease	15 (9.3%)	4 (8.2%)	11 (9.7%)	0.751
Other	15 (9.3%)	7 (14.3%)	8 (7.1%)	0.152
Fever	138 (85.2%)	41 (83.7%)	97 (85.8%)	0.721
Cough	130 (80.2%)	37 (75.5%)	93 (82.3%)	0.319
Dyspnoea	39 (24.1%)	13(26.5%)	26 (23%)	0.63
Pharynalgia	39 (24.1%)	14 (28.6%)	25 (22.1%)	0.378
Thoracalgia	47 (29%)	16 (32.7%)	31 (27.4%)	0.501
Myalgia	50 (30.9%)	15 (30.6%)	35 (31%)	0.964
Nausea	29 (17.9%)	10 (20.4%)	19 (16.8%)	0.584
Vomiting	19 (11.7%)	5 (10.2%)	14 (12.4%)	0.691
Diarrhea	35 (21.6%)	12 (24.5%)	23 (20.4%)	0.557
**Laboratory findings**				
White blood cell count, ×10^9^ per L	5.505 (4.5475–6.85)	5.94 (3.755–8.345)	5.95 (4.9375–7.1925)	0.83
Lymphocyte count, ×10^9^ per L	1.32 (0.995–1.69)	1.21 (0.895–2)	1.355 (1.0875–1.7875)	0.851
Hemoglobin, g/L	125 (113–134.5)	123 (110–126)	126.5 (113~133)	0.1
Platelet count, ×10^9^ per L	246 (201.5–328)	274 (196–331)	238 (212.25–323.25)	0.633
ALT, U/L	21 (14–34)	26 (13–42)	24 (13.75–36.75)	0.594
Lactate dehydrogenase, U/L	36 (31.7–39.8)	35.4 (30.95–39.5)	34.2 (30.725–38.5)	0.505
Albumin, g/L	67 (56–80)	64 (57.5–69.5)	72 (55.75–87.5)	0.541
Creatinine, μmol/L	76.55 (33–188)	99 (40.5–341.5)	94 (48.75–208)	0.201
C-reactive protein, mg/L	6.3 (1.6~31)	14 (2.2–36.1)	7.5 (2.475–38.625)	0.696
NT-pro BNP, pg/ml	0.67 (0.33–1.29)	0.8 (0.59–1.56)	0.72 (0.355–1.8175)	0.723
D-dimer, μg/ml	5.21 (2.965–10.59)	3.87 (3.045–9.78)	5.51 (2.7975–10.825)	0.824
IL-6, pg/ml	232.5 (186–302.75)	251 (205.5–317)	246.5 (208–314.5)	0.756
**Chest CT imaging features**				
Consolidation	19 (12.3%)	6 (13%)	13 (12%)	0.862
Patchy opacity and ground-glass opacity	138 (89.6%)	44 (95.7%)	94 (87%)	0.151
Bilateral pulmonary infiltration	137 (85.1%)	45 (91.8%)	92 (82.1%)	0.112
**Medication history**				
Antiviral treatment	162 (100%)	49 (100%)	113 (100%)	–
Antibacterial treatment	120 (74.1%)	37 (75.5%)	83 (73.5%)	0.784
Corticosteroid treatment	24 (14.8%)	9 (18.4%)	15 (13.3%)	0.402
Traditional Chinese medicine prescription	126 (77.8%)	37 (75.5%)	89 (78.8%)	0.648
Immunoglobulin and/or thymosin treatment	29 (17.9%)	10 (20.4%)	19 (16.8%)	0.584

### Comparison of LHQW Monotherapy and LHQW + Arbidol Combination Therapy in Patients With Severe COVID-19

**Figure 3:**
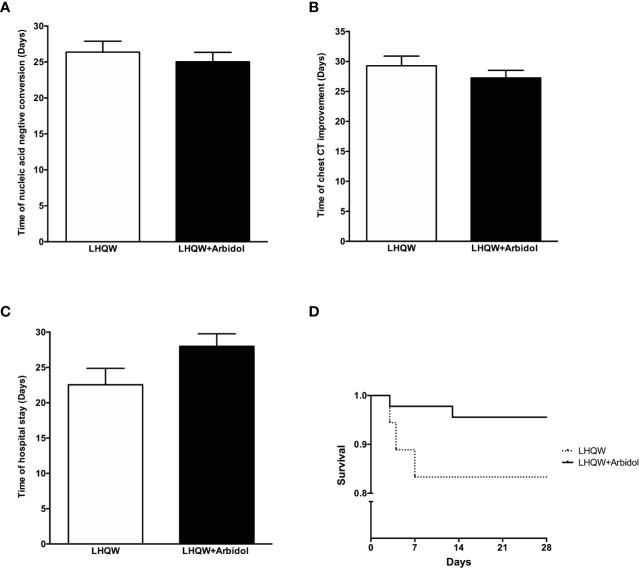
Efficacy of LHQW and LHQW + Arbidol treatment in patients with severe COVID-19. **(A)** Effect on the time to conversion to nucleic acid negativity. **(B)** Effect on time to chest CT improvement. **(C)** Effect on length of hospital stay. **(D)** Comparison of Kaplan–Meier survival curves in patients with severe COVID-19 treated with LHQW and LHQW + Arbidol.

A total of 63 patients with severe COVID-19 were included in this analysis. The detailed analysis of the demographic, clinical, laboratory, and radiographic characteristics and the medication history of all patients with severe COVID-19 is summarized in **Table 2**. Except for significant differences between sexes, there were no significant differences in the demographic, clinical, laboratory or radiographic characteristics or the medication history of patients with severe COVID-19 between the LHQW monotherapy group and the LHQW **+** Arbidol combination therapy group. The sex-based differences may be due to the relatively small sample size of female severe COVID-19 patients who were treated with LHQW monotherapy. We also found that there were no significant differences in the time to conversion to nucleic acid negativity, time to chest CT improvement, duration of hospital stay or Kaplan-Meier survival curves between the LHQW group and the LHQW **+** Arbidol group (**Figure 3**).

**Table 2:** Demographic, clinical, laboratory, and radiographic characteristics and the medication history of patients with severe COVID-19.

	Total	LHQW	LHQW + Arbidol	p value
	(n = 63)	(n = 18)	(n = 45)	
**Demographic and clinical characteristics**				
Age	65 (55.5–74.5)	64 (49–74)	66 (55.75–76)	0.71
Sex	–	–	–	0.021
Female	32 (50.8%)	5 (27.8%)	27 (60%)	–
Male	31 (49.2%)	13 (72.2%)	18 (40%)	–
Hypertension	30 (47.6%)	7 (38.9%)	23 (51.1%)	0.38
Diabetes	11 (17.5%)	3 (16.7%)	8 (17.8%)	0.341
Chronic obstructive lung disease	6 (9.5%)	3 (16.7%)	3 (6.7%)	0.341
Coronary heart disease	7 (11.1%)	2 (11.1%)	5 (11.1%)	1
Other	6 (9.5%)	3 (16.7%)	3 (6.7%)	0.341
Fever	51 (81%)	13 (72.2%)	38 (84.4%)	0.299
Cough	52 (82.5%)	16 (88.9%)	36 (80%)	0.489
Dyspnoea	10 (15.9%)	4 (22.2%)	6 (13.3%)	0.383
Pharynalgia	13 (20.6%)	4 (22.2%)	9 (20%)	1
Thoracalgia	16 (25.4%)	4 (22.2%)	12 (26.7%)	1
Myalgia	22 (34.9%)	6 (33.3%)	16 (35.6%)	0.867
Nausea	14 (22.2%)	5 (27.8%)	9 (20%)	0.517
Vomiting	9 (14.3%)	2 (11.1%)	7 (15.6%)	1
Diarrhea	14 (22.2%)	5 (27.8%)	9 (20%)	0.517
**Laboratory findings**				
White blood cell count, ×10^9^ per L	5.9 (4.81–8.065)	7 (3.81–12.27)	5.69 (4.815–7.145)	0.346
Lymphocyte count, ×10^9^ per L	1.16 (0.802–1.355)	1.08 (0.71–1.89)	1.165 (0.816–1.32)	0.692
Hemoglobin, g/L	124 (110.5–130)	124 (110–128)	123.5 (111.75–132.25)	0.152
Platelet count, ×10^9^ per L	233 (203.5–329)	304 (220–331)	227.5 (201.5–297.75)	0.494
ALT, U/L	26 (13–41.5)	24 (12–42)	26.5 (13–41.25)	0.261
Lactate dehydrogenase, U/L	31.6 (28.8–35.35)	30.4 (28.6–34.5)	32 (29.15–36)	0.945
Albumin, g/L	75 (55.5–95.5)	60 (50–68)	80.5 (60.75–96)	0.115
Creatinine, μmol/L	139 (64.5–495)	408 (58–961)	129.5 (70–289)	0.484
C-reactive protein, mg/L	22.4 (5.95–78.4)	14.5 (9.4–84.7)	27.2 (4.55–73.475)	0.497
NT-pro BNP, pg/ml	1.67 (0.75–2.54)	2.1 (0.95–2.95)	1.245 (0.69–2.365)	0.585
D-dimer, μg/ml	6.81 (3.765–23.63)	7.6 (3.87–49.97)	6.72 (3.655–21.5575)	0.98
IL-6, pg/ml	290 (225.5–358.5)	314 (249–366)	265 (222.75–356)	0.944
**Chest CT imaging features**				
Consolidation	8 (13.1%)	2 (11.8%)	6 (13.6%)	1
Patchy opacity and ground-glass opacity	57 (93.4%)	16 (94.1%)	41 (93.2%)	1
Bilateral pulmonary infiltration	53 (84.1%)	16 (88.9%)	37 (82.2%)	0.71
**Medication history**				
Antiviral treatment	63 (100%)	18 (100%)	45 (100%)	–
Antibacterial treatment	47 (74.6%)	14 (77.8%)	33 (73.3%)	1
Corticosteroid treatment	14 (22.2%)	5 (27.8%)	9 (20%)	0.517
Traditional Chinese medicine prescription	57 (90.5%)	15 (83.3%)	42 (93.3%)	0.341
Immunoglobulin and/or thymosin treatment	14 (22.2%)	5 (27.8%)	9 (20%)	0.517

### Comparison of LHQW Monotherapy and LHQW + Arbidol Combination Therapy in Patients With Moderate COVID-19

**Figure 4:**
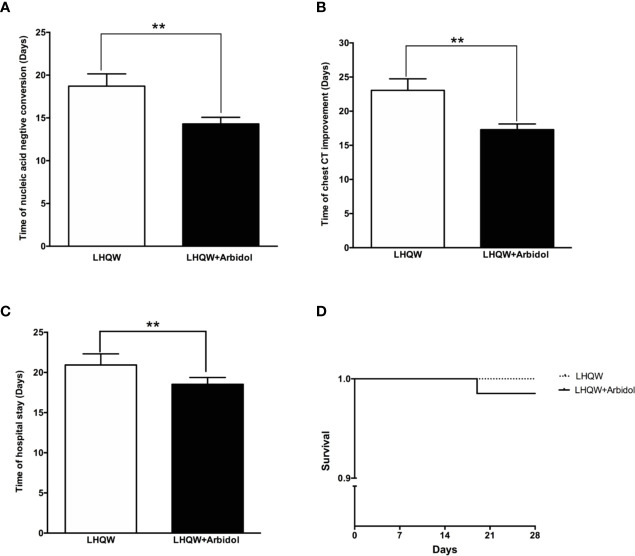
Efficacy of LHQW and LHQW+ Arbidol treatment in patients with moderate COVID-19. **(A)** Effect on the time to conversion to nucleic acid negativity. **(B)** Effect on time to chest CT improvement. **(C)** Effect on length of hospital stay. **(D)** Comparison of Kaplan–Meier survival curves between patients with moderate COVID-19 treated with LHQW and LHQW + Arbidol. **P < 0.01.

A total of 99 patients with moderate COVID-19 were included in this analysis. The detailed analysis of the demographic, clinical, laboratory, and radiographic characteristics and the medication history of all patients with moderate COVID-19 is summarized in **Table 3**. The demographic, clinical, laboratory, and radiographic characteristics and the medication history of patients with moderate COVID-19 were not significantly different between the LHQW monotherapy group and the LHQW**+** Arbidol group. The time to conversion to nucleic acid negativity, time to chest CT improvement, and length of hospital stay in the LHQW **+** Arbidol combination therapy group were significantly shorter than those in the LHQW monotherapy group. However, there were no significant differences in the Kaplan–Meier survival curves (**Figure 4**).

**Table 3:** Demographic, clinical, laboratory, and radiographic characteristics and the medication history of patients with moderate COVID-19.

	Total	LHQW	LHQW + Arbidol	p value
	(n = 99)	(n = 31)	(n = 68)	
**Demographic and clinical characteristics**				
Age	63 (54–70)	67 (57.25–74.25)	59.5 (54–67.5)	0.213
Sex	–	–	–	0.815
Female	43 (43.4%)	14 (45.2%)	29 (42.6%)	–
Male	56 (56.6%)	17 (54.8%)	39 (57.4%)	–
Hypertension	34 (34.3%)	12 (38.7%)	22 (32.4%)	0.537
Diabetes	11 (11.2%)	4 (12.9%)	7 (10.4%)	0.738
Chronic obstructive lung disease	4 (4%)	1 (3.2%)	3 (4.4%)	1
Coronary heart disease	8 (8.1%)	2 (6.5%)	6 (8.8%)	1
Other	9 (9.1%)	4 (12.9%)	5 (7.4%)	0.455
Fever	87 (87.9%)	28 (90.3%)	59 (86.8%)	0.748
Cough	79 (79.8%)	22 (71%)	57 (83.8%)	0.14
Dyspnea	29 (29.3%)	9 (29%)	20 (29.4%)	0.969
Pharynalgia	26 (26.3%)	10 (32.3%)	16 (23.5%)	0.36
Thoracalgia	31 (31.3%)	12 (38.7%)	19 (27.9%)	0.284
Myalgia	28 (28.3%)	9 (29%)	19 (27.9%)	0.911
Nausea	15 (15.2%)	5 (16.1%)	10 (14.7%)	1
Vomiting	10 (10.1%)	3 (9.7%)	7 (10.3%)	1
Diarrhea	21 (21.2%)	7 (22.6%)	14 (20.6%)	0.822
**Laboratory findings**				
White blood cell count, ×10^9^ per L	6.07 (4.69–7.165)	5.35 (3.6175–7.04)	6.31 (5.365–7.2175)	0.323
Lymphocyte count, ×10^9^ per L	1.64(1.195–2.005)	1.27(0.9925–2.0175)	1.665 (1.3875–2.0025)	0.353
Hemoglobin, g/L	124 (116.5–132)	120 (110.25–125)	128 (119–134.5)	0.278
Platelet count, ×10^9^ per L	267.5 (220–335.75)	272 (169.25–340)	255.5 (221.75–341.25)	0.247
ALT, U/L	22 (14.75–36.25)	26 (13.75–47)	21 (15.25–33.75)	0.845
Lactate dehydrogenase, U/L	36.1 (33.25–40.125)	37.05 (34.4–39.85)	35.8 (32.5–41.225)	0.452
Albumin, g/L	65.5 (55.75–77.75)	67 (62.75–72.5)	61 (55–79.25)	0.279
Creatinine, μmol/L	80.5 (28.75–181.5)	75.5 (31.75–193.75)	80.5 (28.25–171.25)	0.429
C-reactive protein, mg/L	3.45 (1.275–20.575)	4.6 (1.6–31.35)	3.45 (1.125–14.7)	0.628
NT-pro BNP, pg/ml	0.555 (0.28~0.8175)	0.685 (0.3175–0.8025)	0.44 (0.26–0.8625)	0.847
D-dimer, μg/ml	3.62(2.47–7.3475)	3.385(2.6475–7.9175)	3.66 (2.43–7.1725)	0.869
IL-6, pg/ml	227.5 (175.75–276)	233 (193.25–287)	226.5 (173.25–274.25)	0.891
**Chest CT imaging features**				
Consolidation	11 (11.8%)	4 (13.8%)	7 (10.9%)	0.735
Patchy opacity and ground-glass opacity	81 (87.1%)	28 (96.6%)	53 (82.8%)	0.096
Bilateral pulmonary infiltration	84 (85.7%)	29 (93.5%)	55 (82.1%)	0.214
**Medication history**				
Antiviral treatment	99 (100%)	31 (100%)	68 (100%)	–
Antibacterial treatment	73 (73.7%)	23 (74.2%)	50 (73.5%)	0.944
Corticosteroid treatment	10 (10.1%)	4 (12.9%)	6 (8.8%)	0.72
Traditional Chinese medicine prescription	71 (71.7%)	24 (77.4%)	47 (69.1%)	0.395
Immunoglobulin and/or thymosin treatment	15 (15.2%)	5 (16.1%)	10 (14.7%)	1

### Efficacy of Early and Timely LHQW+ Arbidol Combination Therapy in Patients With Moderate COVID-19

**Figure 5:**
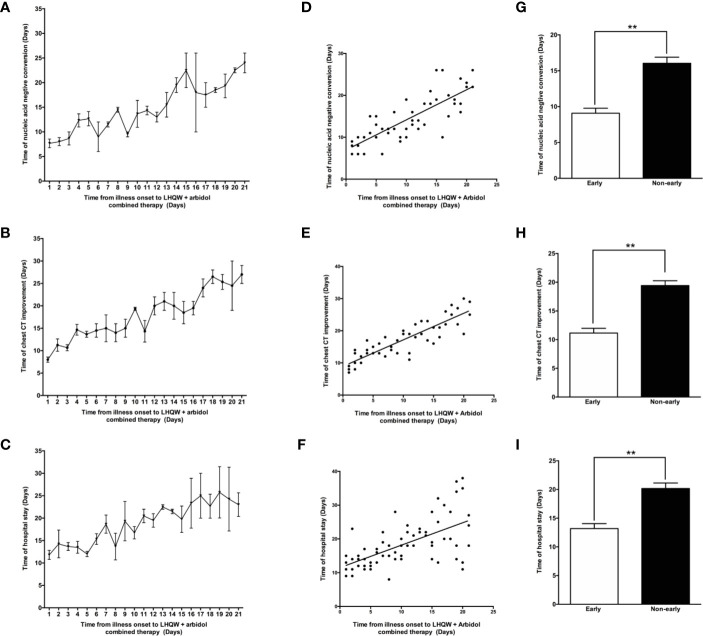
Efficacy of early LHQW + Arbidol combination therapy in patients with moderate COVID-19. The relationships between the time from illness onset to the administration of LHQW **+** Arbidol combination therapy and time to conversion to nucleic acid negativity **(A)**, time to chest CT improvement **(B)**, and length of hospital stay **(C)**. Correlation analysis of time from illness onset to the administration of LHQW + Arbidol combination therapy with the time to conversion to nucleic acid negativity **(D)**, time to chest CT improvement **(E)**, and length of hospital stay **(F)**. The comparison of time to conversion to nucleic acid negativity **(G)**, time to chest CT improvement **(H)**, and length of hospital stay **(I)** between early and non-early LHQW + Arbidol combination therapy administration. **P < 0.01.

As shown in **Figure 4**, LHQW **+** Arbidol combination therapy is more effective than LHQW monotherapy in moderate COVID-19 patients. With the aim of exploring the role of the time to the initiation of combination therapy on treatment efficacy in moderate COVID-19 patients, we analyzed the time to conversion to nucleic acid negativity, time to chest CT improvement and length of hospital stay in moderate COVID-19 patients treated with LHQW + Arbidol combination therapy. The results indicated that if moderate COVID-19 patients were treated with LHQW **+** arbidol soon after disease onset, the average times to throat swab conversion to nucleic acid negativity and chest CT improvement were approximately 8 days, and the average length of hospital stay was reduced to approximately 12 days. With the delayed administration of LHQW + Arbidol, the times to the conversion to nucleic acid negativity and clinical and radiographic improvement were prolonged. The period from illness onset to the administration of LHQW + Arbidol was positively correlated with the time to conversion to nucleic acid negativity (Y = 0.7189 * X + 6.958, r^2^ = 0.660, p < 0.001), time to chest CT improvement (Y = 0.8263 * X + 8.905, r^2^ = 0.770, p < 0.001), and duration of hospital stay (Y = 0.6644 * X + 11.49, r^2^ = 0.374, p < 0.001). The time to conversion to nucleic acid negativity, time to chest CT improvement, and length of hospital stay were significantly reduced in the early LHQW **+** Arbidol combination therapy group compared with the non-early group (**Figure 5**).

## Discussion

This retrospective study identified the effectiveness of LHQW + Arbidol combination therapy for the treatment of COVID-19. The combined usage of Arbidol and LHQW significantly shortened the time to conversion to nucleic acid negativity, time to chest CT improvement and length of hospital stay in patients with moderate COVID-19 but not in those with severe COVID-19. The earlier the administration of LHQW + Arbidol combination therapy was, the faster the recovery of patients with moderate COVID-19. Of note, all enrolled patients recovered and met the criteria for discharge except non-survivors. In this study, patients exhibited excellent tolerance of Arbidol and LHQW. Therefore, no obvious drug-related side effects were observed.

SARS-CoV-2 is an enveloped RNA virus from the Betacoronavirus genus that is distributed in humans and animals, including birds and mammals. A close link between SARS-CoV-2 and a bat coronavirus was discovered at the beginning of the COVID-19 outbreak in Wuhan, China (Zhu N. et al., 2020). The virus targets human cells mainly through its viral structural spike protein that binds to the angiotensin-converting enzyme 2 (ACE2) receptor (Hoffmann et al., 2020). Once SARS-CoV-2 enters the body, a high viral load develops in the upper respiratory tract, and there is potential for asymptomatic patients to shed and transmit the virus (Doremalen, 2020). Due to similarities in the epidemiology, genomics, and pathogenesis of SARS-CoV-2, SARS-CoV-1 and Middle East respiratory syndrome coronavirus MERS-CoV, drugs previously used to inhibit SARS and MERS are potential candidates for the treatment of COVID-19; these drug include antiviral drugs and TCM. The most promising drugs that have been repurposed for COVID-19 have been under investigation since the start of the pandemic (Sanders et al., 2020). Approximately 300 active trials are underway, including an RCT of Arbidol and LHQW in China. Arbidol, a broad-spectrum antiviral, has been licensed in Russia and China mainly for the prevention and treatment of influenza and other respiratory viral infections, and no major adverse effects have been reported. In fact, evidence derived from numerous studies has indicated that Arbidol has extensive antiviral activity *in vitro* and *in vivo* against a number of enveloped and non-enveloped RNA and DNA viruses, including influenza viruses A, B and C; hepatitis viruses B and C; Ebola virus; chikungunya virus; hantavirus; reovirus; respiratory syncytial virus; human rhinovirus type 14; coxsackie virus B3; adenovirus type 7; and SARS-CoV (Shi et al., 2007; Blaising et al., 2014; Pecheur et al., 2016). It was found that Arbidol can effectively inhibit the replication of SARS-CoV-2 at a concentration of 10–30 μM (Global Times, 2020). However, the exact mechanism by which Arbidol inhibits SARS-CoV-2 has not been reported. According to previous studies, arbidol may bind and interact with both viral lipids and protein residues to affect several stages in the viral life cycle, such as entry, fusion, replication, assembly and budding (Teissier et al., 2011). In influenza A virus-infected mice, Arbidol significantly suppressed the levels of IL-1*β*, IL-6, IL-12, and TNF-α and elevated the level of IL-10 in bronchoalveolar lavage fluids and lung tissues, which suggested that Arbidol has an anti-inflammatory effect (Liu et al., 2013). LHQW was approved by the National Medical Products Administration and absorbed in Chinese pharmacopoeia. It is composed of 13 herbs as shown in **Table 4** (Ding et al., 2017) and usually used for the prophylaxis and treatment of viral influenza. LHQW capsules went through strict quality control and supervision in accordance with Pharmacopoeial requirements and standards before leaving the factory. Li and his colleagues found that the similarities in 10 batches of LHQW capsules sample were above 0.96 (Runfeng et al., 2020). 61 components have been isolated and identified form LHQW using rapid ultraperformance liquid chromatography coupled with diode-array detector and quadrupole time-of-flight mass spectrometry (UPLC-DAD-QTOF-MS) method. These identified components were further divided into six types by structural characteristics containing phenylpropanoids, anthraquinones, triterpenoids, iridoids, and other types. Salidroside, chlorogenic acid, forsythoside, cryptochlorogenic acid, amygdalin, sweroside, hyperin, rutin, forsythoside A, phillyrin, rhein, and glycyrrhizic acid are the 12 representative compounds. The total concentration of these 12 representative components in 10 batches of LHQW capsules varied narrowly (Jia et al., 2015). Then 15 main effective components including (arctiin, emodin, formononetin, forsythoside A, gallic acid, hesperidin, isoliquiritigenin, kaempferol, ononin, phillyrin, quercetin, rutin, salidroside, secoxyloganin, and tricin) were identified along with 61 corresponding targets by comparing compound-effective target network and compound-ineffective target network (Wang et al., 2016). Recently, the novel therapeutic application of LHQW to the treatment of COVID-19 has already been added to the indications for LHQW, which were approved by the China Food and Drug Administration. Given its relatively lower price and easier access, a large number of Chinese individuals have chosen to use LHQW to prevent or treat COVID-19.

**Table 4:** Composition of LHQW.

Plant	Family	Weight	Used part
*Forsythia suspensa* (Thunb.) Vahl	Oleaceae	255 g	Fructus
*Ephedra sinica* stapf	Ephedraceae	85 g	Stem
*Lonicera japonica* Thunb.	Caprifoliaceae	255 g	Flower bud
*Isatis indigotica* Fortune	Brassicaceae	255 g	Root
*Mentha haplocalyx* Briq.	Mentha	75 g	Menthol
*Dryopteris crassirhizoma* Nakai	Dryopteridaceae	255 g	Rhizoma
*Rhodiola rosea* L.	Crassulaceae	85 g	Rhizoma
Gypsum Fibrosum	–	255 g	–
*Pogostemon cablin* (Blanco) Benth.	Labiatae	85 g	Whole plant
*Rheum palmatum* L.	Polygonaceae	51 g	Rhizoma
*Houttuynia cordata* Thunb.	saururaceae	255 g	Whole plant
*Glycyrrhiza uralensis* Fisch.	Leguminosea	85 g	Rhizoma
*Armeniaca sibirica* (L.) Lam.	Rosaceae	85 g	Seed

Note: This table was adapted from Ding et al. (2017), doi: 10.1186/s12906-017-1585-7.

Therefore LHQW may have played a positive role in preventing and treating COVID-19, especially in patients with mild symptoms (Yao and Li, 2020). In randomized, double-blind, positive controlled clinical trials, LHQW treatment significantly reduced the severity of illness and the duration of symptoms, including fever, cough, sore throat and fatigue, compared with the outcomes in the oseltamivir group (Duan ZP et al., 2011; Zhao P et al., 2014; Hu et al., 2020) in patients with influenza A virus infection. In an *in vitro* study, LHQW significantly inhibited the replication of SARS-CoV-2, affected virus morphology, and exerted anti-inflammatory effects (Runfeng et al., 2020). LHQW has been shown to have a broad spectrum of effects on various influenza viruses (such as H7N9 and HIN1), which include inhibiting viral proliferation and impacting immune function by suppressing the virus-induced gene expression of NF-*κ*B and alleviating the virus-induced gene expression of TNF-α, IP-10, MCP-1, IL-6, and IL-8 (Ding et al., 2017). In addition, LHQW significantly decreased the release of inflammatory mediators such as IL-8, TNF-α, IL-17, and IL-23 in the sputum and IL-8 and IL-17 in the blood in patients with acute exacerbation of chronic obstructive pulmonary disease (Dong et al., 2014). Our study is the first to report the efficacy of LHQW + Arbidol combination therapy for the treatment of moderate COVID-19. However, our study did not include patients who were treated with Arbidol monotherapy. Therefore, we could not precisely evaluate the contributions of LHQW and Arbidol in the group administered the combination therapy. This is one limitation of our retrospective study. Owing to its efficacy, Arbidol combined with LHQW may be an alternative choice for the treatment of COVID-19. According to previous studies, antiviral, anti-inflammatory and immunomodulatory functions are probably the common mechanisms underlying the effectiveness of LHQW + Arbidol combination therapy for the treatment of COVID-19, although the exact mechanism still needs to be further studied. Due to the limitations of retrospective studies, randomized comparative studies are still needed to further test the effects and advantages of this therapy for COVID-19.

In addition to the efficacy of LHQW + Arbidol combination therapy in patients with moderate COVID-19, we also found that the appropriate time to initiate drug treatment is as early as possible after disease onset. The recovery time is positively correlated with the duration from illness onset to the initiation of LHQW + Arbidol treatment. Another retrospective study also found that early and timely antiviral treatment in mild COVID-19 patients may significantly slow the disease progression and improve the prognosis of patients (Wu et al., 2020). The reason for the variation in the interval from illness onset to the initiation of LHQW **+** Arbidol therapy is that a large number of people were infected with SARS-CoV-2 during the period in which medical resources were relatively limited at the beginning of the COVID-19 outbreak. Based on the results of this study, early treatment with of LHQW + Arbidol combination therapy is beneficial in moderate COVID-19 patients.

In conclusion, this retrospective study demonstrated that the early administration of LHQW + Arbidol combination therapy could significantly accelerate recovery in patients with moderate COVID-19 by reducing the time to conversion to nucleic acid negativity, the time to chest CT improvement and the length of hospital stay. Therefore, we recommended the early and timely administration of the combination of Arbidol and LHQW to accelerate recovery and improve the prognosis in patients.

## Data Availability Statement

All datasets presented in this study are included in the article/supplementary material.

## Ethics Statement

The studies involving human participants were reviewed and approved by the Research Ethics Commission of Ruijin Hospital (KY2020-86). The patients provided their written informed consent to participate in this study.

## Author Contributions

WC, G-CS, and X-LB contributed to the study conception and design. JF and HL contributed to the acquisition, analysis, interpretation of data, and drafting of the paper. WD, PY, Y-YG and S-YM contributed to the review and collection of data. All authors contributed to the article and approved the submitted version.

## Funding

This study was supported by Shanghai Jiao Tong University of Medicine Special Scientific Research Fund for Pharmacy Administration and rational drug use of COVID-19 (JDYX2020KYZX011).

## Conflict of Interest

The authors declare that the research was conducted in the absence of any commercial or financial relationships that could be construed as a potential conflict of interest.
